# Comparing Constant and Transient Membrane Transport Parameters for Use in Wave Desalination Models

**DOI:** 10.3390/membranes15080243

**Published:** 2025-08-09

**Authors:** Kurban A. Sitterley, Zachary Binger, Dale Scott Jenne

**Affiliations:** National Renewable Energy Laboratory, Golden, CO 80401, USAdale.jenne@nrel.gov (D.S.J.)

**Keywords:** desalination, reverse osmosis, wave energy, sustainability

## Abstract

Directly pressurizing seawater for desalination with reverse osmosis membranes via wave motion is a promising and sustainable method for producing freshwater in coastal regions. However, such a system could result in significant pressure fluctuations and a departure from conventional steady-state desalination operations. This study sought to assess if membrane transport parameters (apparent water and salt permeability) should be modeled as transient or constant in solution–diffusion-based modeling efforts of dynamically operated desalination systems, such as those coupled to wave power. Two approaches were used to model membrane transport parameters: one considered each parameter to be a function of the net driving pressure of the system, and the other assumed they were constant across all conditions. A pilot-scale system was used to conduct steady-state and controlled ramping experiments. Data from steady-state experiments were used to calculate transient and constant transport parameters. Parameter combinations were used in a simulation model to predict water flux and effective permeate salinity, and simulation outcomes were compared against experimental ramping results. The transient relationships for both water and salt permeability produced the most accurate results for water flux and comparable results for effective permeate salinity. Development of such relationships would be unique to a specific system but could be valuable in modeling wave-driven desalination systems across the wide range of operating conditions they experience.

## 1. Introduction

A central challenge for society in the 21st century is the reliable availability of both freshwater and energy. As demands for freshwater continue to rise, existing water infrastructure may become increasingly strained due to long-term fuel availability and rising operational costs. As a result, there is a growing interest in more integrated and efficient energy–water systems that can meet future water needs in a sustainable and cost-effective manner. Desalination—the process of removing salt and other components from seawater and brackish water—involves many individual technologies, with the most mature and widely used being reverse osmosis (RO), accounting for 88% of membrane-based desalination processes [1]. Depending on the influent salinity, flow rate, and desired system recovery, the specific energy consumption of a seawater RO system is typically between 2.5 and 7 kWh m^−3^, with larger plants registering at the lower end of that range due to economies of scale [2], accounting for approximately 36–44% of the total operational expenditures of a typical seawater RO plant [3]. Seawater desalination has been established as a reliable approach for augmenting freshwater supplies as surface and groundwater supplies dwindle because of overuse and climate change. However, the two main drawbacks of seawater desalination are the relatively high energy demand, and the high salinity byproduct stream generated that must also be managed.

Seawater desalination via membrane-based technologies is expected to play a critical role in addressing the growing global water demand. However, scaling up these technologies will also require additional energy, potentially leading to increased infrastructure costs and resource dependencies. This issue highlights the need for coupling seawater desalination systems with diversified energy sources to reduce operational constraints and enhance long-term resilience. Despite the technological advancements and lower costs of renewable energy and RO systems separately, combining them into an integrated system presents several practical challenges. Frequently referenced is the difficulty in operating a conventionally steady-state process with a dynamic energy supply and the implications this has for both the membrane and pumping system. Though a less common and less developed renewable energy technology, wave energy could be harnessed for desalination either via a standalone process or in a hybrid/combination renewable-energy-powered desalination system. Wave energy has an inherent advantage of being co-located both with a raw water source (seawater) and large population centers and has been demonstrated to be easier to forecast than solar or wind energy [4], possibly easing the challenge of properly co-locating resources. Pressurizing seawater for desalination using wave energy can be performed indirectly—by first converting wave motion to electricity—or by directly pressurizing the water using a wave-energy converter (WEC).

As interest in wave-driven desalination has increased, the design and modeling of the wave system often receives more attention, with many WEC designs for pressurizing water for desalination being developed and some being deployed [5]. However, the design and modeling of the membrane system has received considerably less attention. Although membrane technologies constitute a highly developed and commercialized sector, compared to wave power technologies, membranes are not intended for the conditions they experience in a wave-driven desalination system. Membrane technologies are designed for steady-state operation in controlled environments. This detail and possible techno-economic and performance implications are frequently ignored in wave-driven desalination literature and proposals. The assumption is generally that the membrane system will behave as expected and that steady-state assumptions for modeling the dynamic system are sufficient. As this technology combination matures, it is important to understand the implications of such a dynamic operating regime on critical membrane mass transfer parameters (i.e., water and salt permeability) to correctly understand and predict the performance of these systems from a design and modeling perspective.

Though less common than steady-state modeling approaches, the literature [6,7] provides examples of dynamic RO models for different applications based on the solution–diffusion model—with or without considerations for concentration polarization (CP)—for single-component spiral-wound RO membranes with time-invariant mass transport parameters. These models are frequently developed in the context of improving RO system control to optimize techno-economic outcomes. In the context of wave-driven desalination, a dynamic RO model was developed [8] to investigate the impact on the CP layer, which requires fully developed conditions; this effect was found to diminish under sinusoidal wave conditions compared to steady-state conditions. That work built on prior efforts in the literature [9] that assumed a constant water permeability coefficient to develop a dynamic computational fluid dynamic RO model investigating pulsed pressure changes on system response. There has also been recent work using physical systems to evaluate membrane performance under dynamic conditions. Sitterley, Cath et al. [10] demonstrated a decline of 7.4% in water permeability for a simulated wave-powered desalination system operated for 1770 hr from 500 to 900 psi while maintaining high salt rejection (>99%) under all experimental sinusoidal wave conditions. An extensive experimental regime evaluating the impact of dynamic feed conditions (flow, pressure) on permeate salinity and flow rate, which identified a marginal recovery increase under rectified sinusoidal wave conditions [11], was also recently reported. Previous investigations [12,13] also found improvements in water production rate with no impact on quality.

Although the existing studies are valuable in assessing the robustness of the RO system under these conditions, they make limited assessments of the impact of these conditions on the fundamental mass transport parameters used in models. The pressure waveform input to an actual wave desalination system would involve a full spectrum of conditions, from gradual to sudden increases in pressure depending on the sea conditions. These changes in pressure also come with a change in flow and a general sudden change in the hydrodynamic conditions present in the membrane channel and at the membrane surface. Given that the traditional solution–diffusion model (and accompanying mass transfer coefficients and relationships) was developed for fully developed steady-state conditions, an investigation into how these mass transfer parameters can be impacted by these conditions is warranted.

Previous experimental evaluations of such conditions have considered both ends of the dynamic spectrum: zero ramping (i.e., steady-state conditions) compared against sinusoidal waveforms [14] up to full WEC Simulator (WEC-Sim)-generated waveforms [15,16]. As wave-powered desalination technology is developing, practitioners must develop adequate models of their anticipated system. Membrane transport parameters—water permeability A (LMH psi^−1^) and salt permeability B (LMH)—are critical for properly modeling the membrane subsystem. Evaluating these parameters via a bench- or pilot-scale is commonly performed [17,18,19,20,21]. However, wave-powered systems are different in that both the pressure and flow rate are fluctuating under expected conditions [11,15,22]. The typical approach for determining the transport parameters in part relies on calculation of the mass transfer coefficient k, often estimated with empirical relationships that are a function of the feed flow rate [23]. When determining transport parameters for systems with varying feed pressure and flow, the reliability of such relationships is unclear.

Recent work has been conducted on the impact of the variable inlet condition (feed pressure and flow) on an RO membrane using a pilot system with sine-wave oscillations [10,22] looking at impacts to membrane performance. Other efforts have focused on experimental investigations of coupled WEC and RO systems testing in wave tank [24]. Such studies typically assume constant values for membrane transport parameters. Transport parameter values can be taken from literature [16,25,26], calculated from the details on the membrane technical specification sheet [27], or estimated experimentally [24,28] (notably, [28] estimated A and B experimentally under a narrow band of operating pressures). In any case, these are estimated using steady-state inlet conditions. Additionally, transport parameter estimation using the solution–diffusion model at lower operating pressures (like those that might frequently be experience by a wave desalination system) are different than those estimated at higher pressures [29]. To the authors knowledge, there is no guidance for estimating membrane transport parameters under dynamic or transient conditions.

The objective of this paper is to characterize the transport parameters under a range of steady-state conditions for an RO pilot system capable of dynamic operation using the solution–diffusion model and assess the predictive power of both transient and constant parameters for the system. The work presented here will assess two assumptions:

1. Correlations for apparent transport parameters developed under steady-state conditions can be used under dynamic conditions to predict system performance.
2. Constant transport parameters determined under steady-state conditions can be used under dynamic conditions to predict system performance.

Two sets of parameters are calculated from steady-state experimental data. These parameter values are then used in a solution–diffusion-based simulation model to predict water production and product salinity, and the outcomes are compared against experimental data.

## 2. Materials and Methods

### 2.1. Experimental System

**Figure 1:**
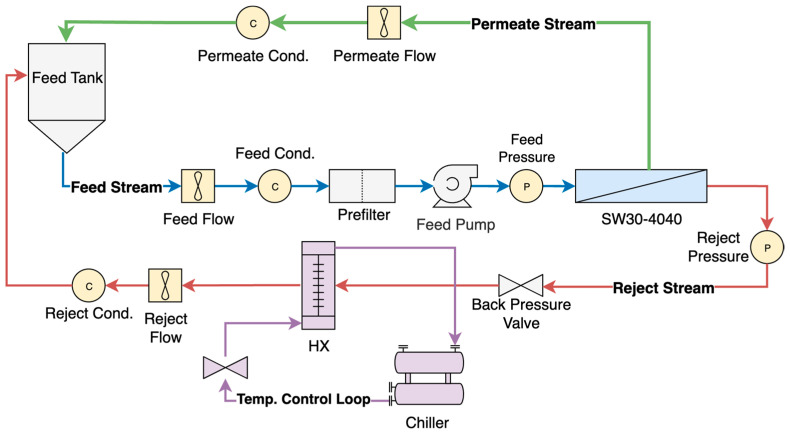
Process flow diagram of experimental system.

The system used to conduct the experiments was similar to that used in prior published work [10]. A process flow diagram of the system is presented in Figure 1. Feed water flows by gravity from a 70-gallon tank through an in-line flow meter and a conductivity sensor on the pump intake to a positive displacement pump. The Hydra-Cell Pro DX-15 pump (Wanner Engineering, Inc.; Minneapolis, MN, USA) supplies feed water through an in-line pressure sensor to a pressure vessel containing a DuPont FilmTec SW30-4040 RO membrane with an active area of 7.4 m^2^ (DuPont Inc.; Wilmington, DE, USA). Both brine and permeate flows are recycled to the feed tank. The brine stream flows through another in-line pressure sensor, a back-pressure valve, a heat exchanger, a flow meter, and a conductivity sensor before returning to the feed tank. Permeate flows through the flow meter and conductivity sensor before returning to the feed tank. The temperature of the feed stream is measured by a temperature sensor located at the bottom of the feed tank. It is regulated by a flow valve controlling the 50/50 glycol/water solution from the chiller to the heat exchanger via the system control and data acquisition system (LabJack; Englewood, CO, USA). The in-line pressure sensors were calibrated via an in-line analog pressure sensor. Conductivity probes were calibrated with standards relevant to the conductivity range they are intended to measure. Data was collected in 0.25 s intervals during each experiment.

The feed water comprised a dechlorinated tap water and NaCl solution. All experiments were conducted at 20 ± 1 °C. Whereas other systems generate pressure fluctuations via modulating the back-pressure valve, this system regulates pressure by following a user-specified pressure profile via changing the flow rate while keeping the back-pressure valve at a constant closed fraction. This method is preferable to regulating via the back-pressure valve because this configuration mimics the fluctuating flow rates that are more typical of a wave-powered desalination system.

### 2.2. Experimental Conditions

In general, the objective of this work is to calculate apparent water and salt permeability coefficients under steady-state conditions and then apply those to dynamic conditions to assess their predictive power. Thus, two sets of experiments were conducted: steady-state and ramping experiments. We assumed the operational bounds of the system to be 500–900 psi (34.5–62.1 bar) as this represents a practical lower and upper bound for any RO system; 500 psi (34.5 bar) is slightly above the approximate osmotic pressure of seawater, so would still result in some permeate production, and 900 psi (62.1 bar) is near the safe operating limit of the pressure vessels (1000 psi; 68.9 bar) and falls within the range of a conventional RO system. This range of pressures also encompasses operating pressures for simulated wave-driven systems [15,30]. Steady-state experiments were conducted at 100 psi (6.9 bar) intervals spanning this entire pressure range (i.e., 500, 600, 700, 800, and 900 psi; 34.5, 41.4, 48.3, 55.2, and 62.1 bar).

The experimental design was developed to ensure that the ramp rates explored fell within the range of possible ramp rates as presented by a previous wave desalination model developed using WEC-Sim [15,30] and previous wave-driven desalination experiments [10,22]. The ramp rate is the change in hydraulic pressure divided by the change in time (ΔP/Δt). In total, we considered four ranges for the change in hydraulic pressure (ΔP = 100, 200, 300, 400 psi; 6.9, 13.8, 20.7, 27.6 bar)—all having a maximum pressure of 900 psi (62.1 bar)—and seven values for the change in time (Δt = 4, 5, 6, 8, 10, 30, 60 s), for a total of 28 ramping conditions. The waveform for each set of experimental conditions took the form of a trapezoidal wave. Whereas the duration of the ramping portions of each experiment varied, the constant hold durations (i.e., either at 500 or 900 psi; 34.5 or 62.1 bar) were held for 60 s between ramps.

### 2.3. Data Processing

All data processing and analysis was performed in Python 3.10 using custom scripting for this project. Prior to any calculations, the data was passed through a Savitz-Golay filter using the scipy Python package [31,32]. Steady-state results were processed separately from each of the dynamic datasets. Filtering of the steady-state data used a window size equal to 5% of the length of the entire dataset (3 sec for every 60 sec of data). The experimental data used a window size equal to 5% of the length of the dataset for a single trapezoidal wave (e.g., for a 10 s ramp time, the total length of the trapezoidal wave is 60 + 60 + 10 + 10 = 140 s, so a 7 s window is used, corresponding to 28 datapoints at 0.25 s per iteration). In both cases, a third-order polynomial was used to fit the samples.

After data filtering, the raw data collected from the experimental system was used to calculate transport and performance parameters needed to compare the two assumptions. Two approaches were taken to investigate these assumptions:

1. An apparent water permeability ($A^{\prime }$) and salt permeability ($B^{\prime }$) parameter is calculated for each of the steady-state experiments. These values are then regressed as a function of the NDP, enabling calculation of apparent transient coefficients under ramping conditions.
2. A constant value for each parameter is calculated from the entire steady-state experimental dataset.

The following sections describe the approaches taken for determining each set of membrane transport parameters. For both approaches, the membrane element is considered as zero-dimensional (i.e., no consideration is made for changes along the length of the membrane), and the solution–diffusion model is assumed to describe membrane performance [33].

(1) $$J_{w}=A\left(∆P-∆\pi_{m}\right)\;$$

where $J_{w}$ is the water flux (LMH), A is the membrane permeability coefficient (LMH psi^−1^), ΔP and $\Delta \pi_{m}$ are the differences in hydraulic and osmotic pressure (psi) across the membrane, respectively. The NaCl concentration in each steam was calculated using a calibration curve developed with the experimental system:

(2) $$c_{NaCl}=0.6638\kappa \;$$

where κ is the measured conductivity (mS cm^−1^). This salinity was used to calculate the osmotic pressure of each stream [34]:

(3) $$\pi =c_{NaCl}\times 0.741829+{\left(c_{NaCl}\right)}^{2}\times 0.00111004\;$$

Note this equation returns osmotic pressure in bar but was converted to psi for the calculations. Water recovery (Y) is calculated via:

(4) $$Y=\frac{q_{p}}{q_{f}}$$

where $q_{p}$ and $q_{f}$ are the permeate and feed flow rates, respectively (L min^−1^). Assumed parameters for the feed stream and the membrane are provided in Table 1.

#### 2.3.1. Apparent Parameter Calculation

Apparent parameter calculation uses the data collected to calculate an average apparent value for each of the steady-state experiments using typical equations and assumptions. Similar methods and assumptions have been used to calculate these parameters in previous works [18,36,39].

The hydraulic pressure (ΔP) on the feed side of the membrane is assumed to be the average of the measured feed $P_{f}$ and brine $P_{b}$ pressure. Because the permeate channel is open to the atmosphere ($P_{p}=0$), the ΔP or transmembrane pressure (TMP) is:

(5) $$∆P=\frac{P_{f}+P_{b}}{2}\;\;$$

The mass transfer coefficient (*k*) was calculated using the Sherwood number, diffusivity ($D_{NaCl}$), and hydraulic diameter ($d_{h}$) of the membrane channel via Equations (6)–(10) [23,40,41]:

(6) $$d_{h}=\frac{4\varepsilon }{\frac{2}{h_{chan}}+\left(1-\varepsilon \right)\left(\frac{8}{h_{chan}}\right)}\;$$

(7) $$Re=\frac{v_{chan}d_{h}}{\nu }\;$$

(8) $$Sc=\frac{\nu }{D_{NaCl}}$$

The Sherwood number correlation used here [23] assumes the ratio of the distance between parallel filaments of the spacer to the filament diameter (L/D) ratio of 6 and angle between crossing elements of 90°. Many other correlations exist, but evaluation of other relationships is beyond the scope of the present study:

(9) $$Sh=0.14Re^{0.64}Sc^{0.42}$$

(10) $$k=\frac{ShD_{NaCl}}{d_{h}}$$

where $d_{h}$ is the hydraulic diameter (m), $w_{mem}$ is the membrane width (m), $h_{chan}$ is the height of the membrane channel (m), Re is the Reynolds number, Sc is the Schmidt number, Sh is the Sherwood number [40], $D_{NaCl}$ is the diffusivity of NaCl (m^2^ s^−1^), and *k* is the mass transfer coefficient (m s^−1^).

The salt concentration at the membrane surface ($c_{m}$) is estimated using the concentration polarization factor (CPF) [42]:

(11) $$CPF=\exp\left(\frac{J_{w}}{k}\right)=\frac{c_{m}-c_{p}}{c_{f}-c_{p}}=\frac{\Delta c_{m}}{\Delta c_{f}}$$

And the change in osmotic pressure from the bulk to permeate side is assumed to be:

(12) $$\Delta \pi_{b}=\pi_{f}-\pi_{p}\;$$

This term is used in Equation (1) to account for CP:

(13) $$J_{w}=A\left(∆P-\Delta \pi_{b}\left(\exp\frac{J_{w}}{k}\right)\right)$$

The difference in the hydraulic and osmotic pressure on the right side of this equation is termed the net driving pressure (NDP):

(14) $$NDP=∆P-\Delta \pi_{b}\left(\exp\frac{J_{w}}{k}\right)\;$$

Then, according to the solution–diffusion model, salt transport across the membrane can be described by Equation (15):

(15) $$J_{s}=B\Delta c_{m}\;$$

where $J_{s}$ is the salt flux (g m^−2^ s^−1^), B is the salt permeability coefficient (m s^−1^), and $\Delta c_{m}$ is the salt concentration difference across the membrane (g L^−1^), determined from Equation (11) [21]. Salt flux $J_{s}$ is calculated using the measured permeate concentration at the outlet $c_{p}$, permeate flow $q_{p}$, and membrane area $A_{m}$:

(16) $$J_{s}=\frac{c_{p}q_{p}}{A_{m}}$$

Note that though Equation (15) describes localized salt flux along the membrane element (since $\Delta c_{m}$ would vary along the membrane length in fact) and Equation (16) describes average salt flux, these were assumed to be equivalent in these calculations due to the zero-dimensional treatment.

The calculated A value from Equation (13) and B value from Equation (15) are assumed to be the apparent water and salt permeability ($A^{\prime }$ and $B^{\prime }$, respectively). Two curves are developed for the water and salt permeability coefficients as a function of NDP based on the steady-state experiments:

(17) $$A^{\prime }=a_{1}\exp\left(-a_{2}NDP\right)+a_{3}$$

(18) $$B^{\prime }=b_{1}\ln\left(b_{2}NDP\right)+b_{3}$$

NDP was chosen as the independent variable in these equations because the hydraulic pressure ΔP is positively correlated with the measured parameters that are used to estimate NDP via the solution–diffusion equation (see Equation (14)). Due to this linearity, Equation (13) can be solved for A and the apparent value $A^{\prime }$ can be approximated with Equation (17). Similarly, the form of Equation (18) was chosen based on the observed logarithmic relationship between increased pressure and increased flux and rejection [42]. Note that other relationships could be developed for these apparent values based on the assumptions used.

#### 2.3.2. Constant Parameter Calculation

The second approach was recently presented by Li and Li in [43] to calculate transport parameter values using experimental sinusoidal wave-driven desalination data from [22]. The only concentration measurement required is the feed concentration, which is assumed to be constant.

Note that Li and Li use spatially averaged values (i.e., across the length of the membrane element; denoted by overbar) for water flux ($\bar{J_{w}})$ and salt flux ($\bar{J_{s}}$) that we assume are equivalent to those calculated via experimental data (i.e., with the zero-dimensional modeling assumption, all measured and calculated values are de facto spatially averaged). The spatially averaged salt concentration ($\bar{c}$) in the feed channel is:

(19) $$\bar{c}=c_{f}\frac{-\ln\left(1-Y\right)}{Y}$$

where $c_{f}$ is the constant feed concentration. This leads to an alternative formulation of the solution–diffusion equation [43,44]:

(20) $$\frac{\Delta P}{J_{w}}=\frac{\left[-\ln(1-Y)/Y\right]CPF}{J_{w}}\pi_{f}+\frac{1}{A}$$

where the FilmTec equation for the CPF is adopted instead of Equation (11) [27]:

(21) $$CPF=\exp\left(0.7Y\right)$$

A more complete derivation is provided in [43]. From Equation (20), plotting $\Delta P/J_{w}$ versus $[-\ln\left(1-Y)/Y\right]CPF/J_{w}$ yields a straight line with the slope $\pi_{f}$ and the inverse of the intercept corresponding to A, thus enabling calculation of a constant water permeability coefficient for a particular system over a range of conditions.

Assuming cp=JsJw [44], $J_{w}$ can be expressed similarly to Equation (20) in terms of B [43]:

(22) $$\frac{\Delta P}{J_{w}}=\frac{\pi_{f}}{B}+\frac{1}{A}\;$$

Combining Equations (20) and (22) leads to elimination of A:

(23) $$\pi_{p}=B\pi_{f}\left(\frac{[-\ln(1-Y)/Y]CPF}{J_{w}}\right)\;$$

And assuming $\pi_{f}$ is linear with $c_{f}$ (note that Equation (3) can be approximated as linear for salinities in this study):

(24) $$c_{p}=B\left(\frac{c_{f}[-\ln(1-Y)/Y]CPF}{J_{w}}\right)$$

Thus, a plot of $c_{p}$ vs. $c_{f}[-\ln\left(1-Y)/Y\right]CPF/J_{w}$ should yield a straight line passing through the origin with the slope equal to B, enabling calculation of a constant salt permeability value for the system.

### 2.4. Parameter Comparison

The water and salt permeability coefficients determined by each of these approaches are then used in a simulation model using the governing equations of the solution–diffusion model. The feed concentration $c_{f}$, feed flow rate $q_{f}$, and transmembrane pressure TMP were fixed prior to running each simulation. The simulations were run as a series of steady-state models linked together via changing pressure and flow rate. Resulting from the zero-dimensional treatment of the membrane element, temporal changes in the membrane channel was neglected. Similar assumptions were made in [14] for a one-dimensional model, where a pseudo-steady-state condition was justified for water and momentum balances by assuming pressure propagation along the membrane element was nearly instantaneous.

The middle three cycles of each experimental ΔP/Δt combination was simulated using both transient and constant parameters and the comparison is made based on the mean absolute error (MAE) of the instantaneous flux ($J_{w}$) and the percent difference in the effective permeate concentration ($c_{eff}$) against the experimental results. This is the resulting concentration if all the permeate water over a given period of time was directed to a holdup volume. $c_{eff}$ was calculated as the total mass of salt through the membrane divided by the total volume of water through the membrane over the simulated period of time:

(25) $$c_{eff}=\frac{\sum_{0}^{i}J_{s,i}A_{m}\Delta t}{\sum_{0}^{i}q_{p,i}\Delta t}\;$$

## 3. Results and Discussion

### 3.1. Steady-State Experiments

**Figure 2:**
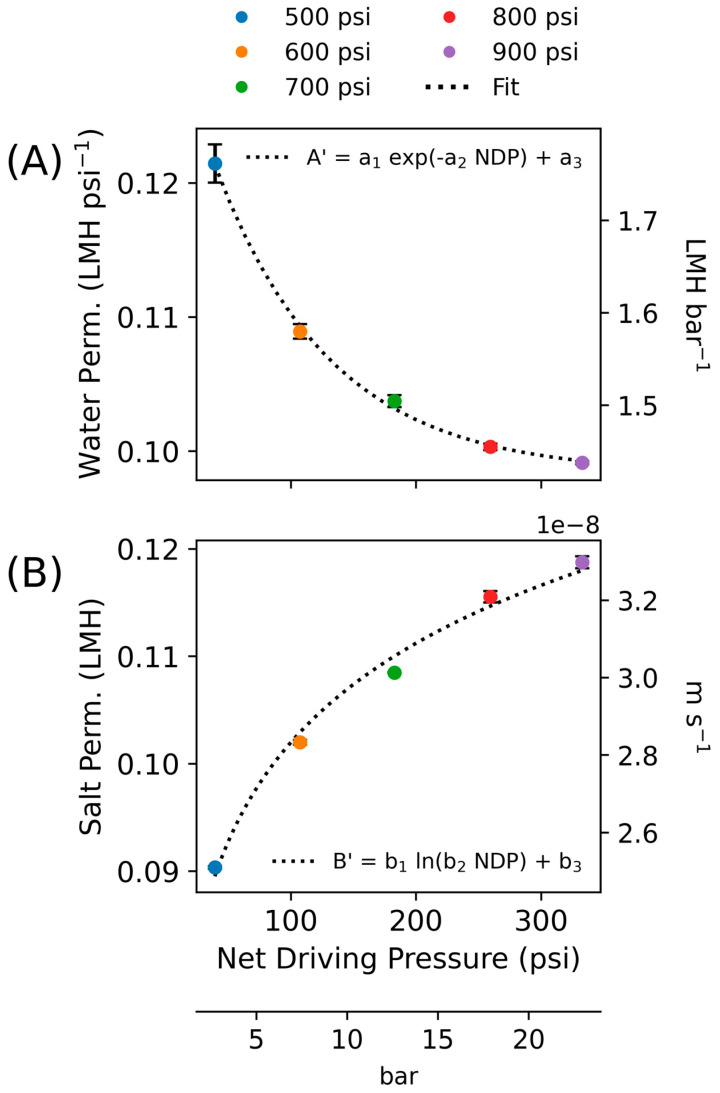
Results of apparent membrane parameters for steady-state experiments (**A**) Apparent water permeability coefficient as a function of NDP fit to a_1_ + exp(-a_2_ NDP) + a_3_; (**B**) Apparent salt permeability coefficient as a function of NDP fit to b_1_ + ln(b_2_ NDP) + b_3_.

We first present steady-state results for apparent values. Using this approach, parameter values were calculated for each time step, and then averaged values were used to fit the general forms in Equations (17) and (18), presented in Figure 2. The calculated values used to make the regression are presented in Table 2; the regression parameters are shown in Table 3. Each parameter showed a strong fit with R^2^ of 99.1% for water permeability and 98.8% for salt permeability across the entire steady-state dataset. The water permeability values ranged between 0.10 and 0.12 LMH psi^−1^ (1.4–1.8 LMH bar^−1^) and salt permeability values ranged between 0.09 and 0.12 LMH, both of which are within the range of other [29] published values for seawater RO membranes (~0.1–0.25 LMH psi^−1^ and 0.03–0.19 LMH for water and salt permeability, respectively; see Figure 5) [18].

Presenting several different “apparent and transient” values for a parameter that is often considered “constant” is unconventional. However, noting that the common formulation of the solution–diffusion model neglects advective transport, the authors of [29] performed an assessment of the errors associated with using the common assumptions and approaches researchers use to determine A and B values for a given system (including some of the same assumptions we used in this work). This required testing across several pressure values, resulting in A vs. ΔP plots that closely resemble the results presented here (starting high and then converging at a constant value with increased pressure). The apparent A value was overestimated by 19–78% at the lowest applied pressure in that work. If applied to the results here, that range of error would suggest the true A value for this system is between 0.100 and 0.068 LMH psi^−1^ (1.45 and 0.99 LMH bar^−1^). Alternatively, significantly increasing the input NDP into the regressed equation presented here (i.e., Equation (17)) results in A = 0.098 LMH psi^−1^ (1.42 LMH bar^−1^). Deviations from the true value in that work were attributed to their assumptions for the CP modulus and membrane swelling (decompaction) at the lowest pressure. Assuming a constant value for the CP modulus, [28] presented A and B values over a narrow band of low operating pressures (~70–120 psi; 5–8 bar) for a 1:10 scaled model of a wave desalination system, showing slightly increasing A, which deviates from the trend observed here, and a linearly increasing B with increasing pressure. Recent work has studied the effect of membrane compaction at higher pressures and the impact on water permeability, suggesting densification of the mesoporous polysulfone layer leads to a longer effective diffusion path for water molecules and a lower water permeability [45,46]. The majority of this water permeability loss is thought to be irreversible, but some fraction has observed to be recovered after “relaxation” periods, an effect attributed to the thermoset property of the polyamide active layer [46]. The effects of relaxation are thought to occur rapidly, with the shortest relaxation period tested in [46] being 3 min, which showed comparable water permeability recovery as 30 min, 60 min, and 24 hr relaxation periods for SW30 membranes. However, the effect of relaxation has only been evaluated under constant pressure regimes, and thus any potential recovered water permeability as a result of lower pressure (not zero pressure) is unknown.

The results from [29] for the B value were also similar to the results presented here, showing increasing salt permeability with increasing pressure and the lowest error relative to the true (or “reference” value, as designated in [29]) value at the lowest applied pressures and higher applied pressures diverging from the true value depending on the assumptions used for the CP modulus. More accurate measurements were observed when the CP modulus was calculated as a function of mass transfer coefficient (resembling the approach used here) rather than using a constant assumption. Notably, this reference value for B was shown to increase with increasing pressure, supporting the trend in this work and indicating that using a transient relationship for wave desalination models would be a more accurate representation of B than assuming it as constant. Increasing B values at higher pressure likely result from increased partitioning of salt into the membrane, resulting in higher salt flux and higher apparent B values [45].

**Figure 3:**
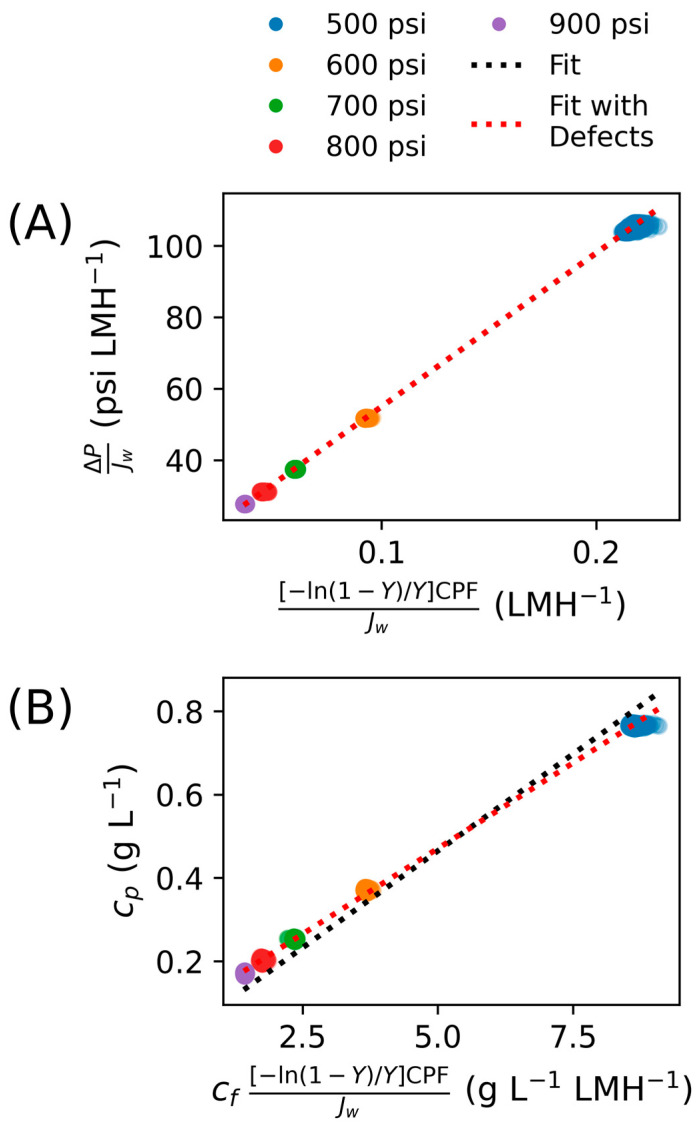
Fit of steady-state data for (**A**) water permeability, where the inverse of the slope corresponds to a constant water permeability, and (**B**) salt permeability, where the slope of the fitted line corresponds to a constant salt permeability. The black dashed line is the fit without defects; the dashed red line is the fit with defects. The resulting A and B values are: without defects, A = 0.083 LMH psi^−1^ (1.20 LMH bar^−1^) and B = 0.093 LMH; with defects, A = 0.083 LMH psi^−1^ (1.20 LMH bar^−1^) and B = 0.082 LMH. Note in panel (**A**) the two fits are indistinguishable from each other.

Figure 3 presents the results of fitting the steady-state dataset to Equations (20) and (24) as described in the methods section to obtain constant values. The water permeability regression R^2^ value was 99.9% and resulted in A  = 0.084 LMH psi^−1^ (1.22 LMH bar^−1^); the salt permeability fit showed an R^2^ value of 97.2% and B = 0.093 LMH. Note that in these calculations, the equation was forced to pass through zero. This constant water permeability is lower than any of the apparent values calculated and lies in the bottom quartile for commercial membranes taken from literature (see Figure 5).

This permeate concentration was also modeled to underpredict at the lower end of the concentration range but overpredict at the upper end (black dashed line in Figure 3B), with MAE of 0.038 g L^−1^ (22.4%). A similar phenomenon was observed in [43], where this was attributed to defects in the membrane surface. According to that work, “defects” could include “minor structural imperfections, such as microvoids, cracks, or pinholes,” a phenomenon originally observed by Eriksson in [47] and applied to modeling osmotic processes in other contexts [48,49,50]. As in [43], an adjustment to account for these defects was made in the modeling approach according to the solution–diffusion-with-defects model [50]:

(26) $$\frac{\Delta P}{J_{w}}=\left(\frac{\pi_{f}[-\ln(1-Y)/Y]CPF}{J_{w}}\right)\left(\frac{1}{1+\beta }\right)\;$$

(27) $$c_{p}=\frac{\bar{c}\left(B\left(CPF\right)+\beta A\left(\Delta P\right)\right)}{J_{w}}\;$$

where β is introduced as the defect ratio, representing the fraction of the active membrane area with defects to the total area. To obtain a linear relationship between B and $c_{p}$ similar to Equation (24):

(28) $$c_{p}=B\frac{c_{f}CPF[-\ln\left(1-Y)/Y\right]}{J_{w}}+\frac{c_{f}\beta A\left(TMP\right)[-\ln\left(1-Y)/Y\right]}{J_{w}}$$

The intercept in this equation could be considered the permeate concentration attributable to the defects, or the “leak” of salt through the membrane defects $c_{leak}$; thus,

(29) $$\frac{c_{leak}}{c_{f}}=\frac{\beta A\left(TMP\right)[-\ln\left(1-Y)/Y\right]}{J_{w}}\;$$

To determine the defect ratio β for this system, a model was developed using the system of equations and solving approach in [43,44]: ΔP and Y were fixed to the experimental values and A, B, β, and $c_{f}$ were left as degrees of freedom. All of the steady-state data was used. An objective was set to minimize the relative discrepancy of the modeled versus experimental values for $c_{p}$ and $J_{w}$:

(30) min A, B,Jw,βf ∑iJw,modelJw,exp−12−cp,modelcp,exp−12

The results for this approach to determining parameter values, termed the “optimized” approach henceforth, are A = 0.083 LMH psi^−1^ (1.20 LMH bar^−1^), B = 0.049 LMH, $c_{f}$ = 37.8 g L^−1^ ($\pi_{f}$ = 429 psi; 29.6 bar) and β = 0.11%. Notably, this optimization resulted in a lower $c_{f}$ than that calculated from the conductivity via Equation (3) (average $c_{f}$ = 39.1 ± 0.3 g L^−1^; average $\pi_{f}$ = 450 ± 3 psi; 31.0 ± 0.2 bar). Such a discrepancy suggests an error with the approach used to calculate the stream concentration from the conductivity. The relationship in Equation (3) is linear across all conductivity values, which is known not to be the case [51], but was a simplification used for processing purposes. When, instead of leaving $c_{f}$ as a degree of freedom, it is fixed to this experimental value, the model pushes the modeled value of B the lower bound that is set, similar to the outcome obtained in [43].

**Figure 4:**
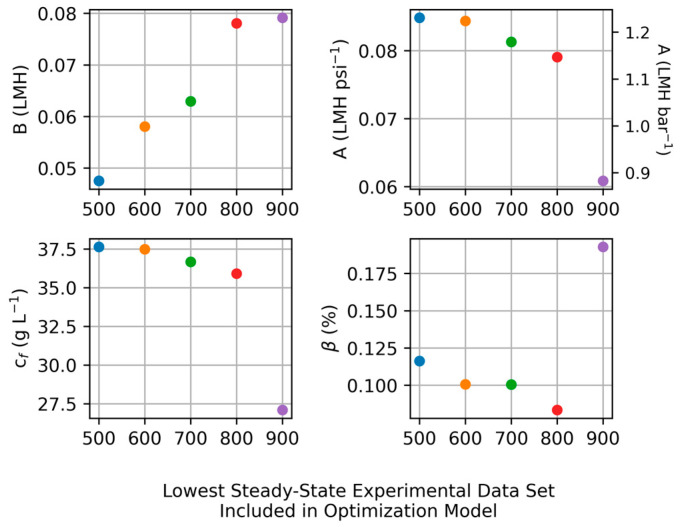
Determination of A, B, β, and $c_{f}$ via optimization method. Horizontal axis represents the lowest pressure of the included datasets. Moving left to right, increasingly fewer datapoints are included in the optimization model.

This optimization-based approach was explored more by observing the impact to the modeled parameters of including more or less data in the model. Figure 4 presents the results of this effort. The x-axis is the value of the pressure for the lowest steady-state experiment included in the model. For example, the values for “500” include all the steady-state datasets (i.e., datasets for 500–900 psi), the values for “600” exclude the 500 psi dataset (i.e., datasets for 600–900 psi), and so on. Going from left to right in Figure 4, increasingly fewer datapoints are included in the optimization model. The values for A, β, and $c_{f}$ appear in good agreement with each other as long as there are at least two datasets included (i.e., values for 500, 600, 700, and 800 in Figure 4; under these conditions, A = 0.083 ± 0.002 LMH psi^−1^ (1.20 ± 0.03 LMH bar^−1^), β = 0.103 ± 0.011%, $c_{f}$ = 37.1 ± 0.6 g L^−1^) while B has more deviation (0.060 ± 0.011 LMH). For the case where the single dataset is included, the results for A, β, and $c_{f}$ deviate significantly from the other results, suggesting limitations to calculating transport parameters using a single experimental condition.

The transport parameters were recalculated via Equations (26) and (28) using the β value determined from the optimization approach and including all the steady-state data, resulting in A = 0.083 LMH bar^−1^ (1.20 LMH bar^−1^), and B = 0.082 LMH. The R^2^ for the water permeability correlation is again high at 99.9% and accounting for defects increases for the salt permeability fit to 99.9%. The intercept for the salt permeability fit is $c_{leak}$ = 0.06 g L^−1^ and the $c_{leak}/c_{f}\;=$ ~0.16%. Incorporating β into the calculation for water permeability was shown not to have a significant impact. For salt permeability, accounting for defects resulted in a 10% reduction in the value obtained from excluding defects; similarly, the optimized approach resulted in a 37% reduction in salt permeability.

The parameter values determined via the steady-state experiments allow testing the two assumptions posited regarding water and salt permeability values under dynamic conditions. To test the first assumption (apparent and transient transport parameters can be used to model dynamic conditions), we developed a relationship with NDP for both $A^{\prime }$ and $B^{\prime }$. To test the second assumption (constant parameters can be used to model dynamic conditions), we obtained three pairs of parameter values using three related methods, each resulting in a single value for A (0.083 LMH psi^−1^; 1.20 LMH bar^−1^) and range of values for B (0.093 LMH excluding defects, 0.083 LMH including defects, and 0.049 LMH from the optimization method). A summary is presented in Table 4.

**Figure 5:**
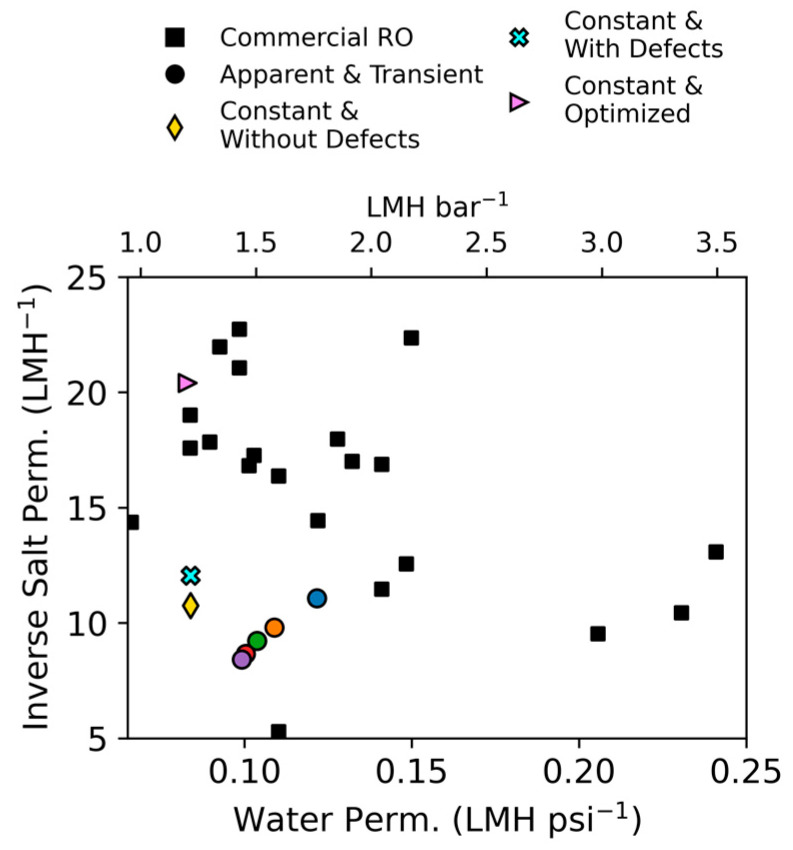
Calculated A vs. 1/B values for each approach used in this work compared against A and 1/B values from commercially available RO membranes. Black squares are values extracted from [18].

The variability in results obtained for these critical parameters underscores the challenge in predicting them experimentally with larger systems under dynamic conditions. These results are plotted in Figure 5 against a dataset of A and B values for commercially available thin-film composite polyamide membranes calculated from conditions on technical spec sheets (data extracted from [18]). All A values calculated here are in the range of the RO values presented in Figure 5 (average A = 0.124 ± 0.046 LMH psi^−1^; 1.80 ± 0.67 LMH bar^−1^). However, one would never obtain a water permeability value derived using the constant assumption by using the equation developed with the apparent and transient assumption. If the results outlined in [29] applied here, we would expect the apparent and transient assumption to converge on the true permeability value at large values for NDP. This result suggests a limitation, error, or incorrect assumption in one of the two approaches, with one possibility being the choice of Sherwood number relationship used to calculate the mass transfer coefficient k (Equation (9)) in the transient approach. The choice of this relationship has direct implications for the calculated $A^{\prime }$ values used to develop the empirical fit. Under the approach used for the constant assumption, k is not calculated and instead a manufacturer-derived relationship to recovery is used to account for CP (Equation (21)). Experimental characterization of the mass transfer coefficient and/or the nature of CP under dynamic conditions can have significant impacts on the parameter values calculated and should be the subject of future experimentation.

With the exception of the optimization-derived value, the salt permeability calculated with both approaches is lower than the majority of the datapoints in Figure 5 (average B = 0.067 ± 0.032 LMH), though still in the same range. However, testing conditions are known to impact calculations of salt permeability, indicating that it is not an intrinsic membrane property as is commonly thought [21,52] and suggesting that comparing the B values determined here to other published values is of limited value. Similarly to the water permeability value, one would never obtain a salt permeability value derived using the constant assumption by using the equation developed with the apparent and transient assumption under the ramping conditions considered here. Like the water permeability coefficient, this could also be related to the approach used to calculate the mass transfer coefficient (related to calculation of B via Equations (11) and (15)), as the crossflow velocity is known to impact the mass transfer coefficient and, consequently, the modeled water flux and salt rejection [42,53]. Proper characterization of the mass transfer coefficient as a function of crossflow velocity for a given system would lead to more accurate A and B calculations [53] and possibly resolve the discrepancies observed.

Discrepancies between the constant salt permeability values and data from Figure 5 could also be related to the steady-state experimental data used to determine the constant salt permeability coefficient via Equation (24) (or Equation (28)). To briefly test this theory, when the 500 psi dataset was excluded from the fit for Equations (24) and (28), the result was B = 0.106 LMH (without defects) and B = 0.089 LMH (with defects). This is a similar result to those obtained via the exploration presented in Figure 4, where the parameter values changed depending on the data that is included. One might expect that the value for an intrinsic property determined with more or less data would be closer in value across determinations; understanding the reasons for these discrepancies should be the subject of future work. Ideally, this would also reveal a proper experimental regime needed to determine transport parameters under these conditions.

Like steady-state desalination systems, proper characterization of water and salt permeability and mass transfer are critical to successful modeling, design, and operation of a wave desalination system [54], particularly under variable conditions [55]. However, the variable feed flow rate (and therefore, crossflow velocity) inherent to wave desalination systems adds additional challenges to accurately modeling the system. Variable temperature can impact water flux and salt rejection [56], and persistent high temperatures have been shown to negatively impact performance [57]. Similarly, higher crossflow velocity will reduce the impact of CP on water flux and salt rejection, but this comes with diminishing returns on recovery due to the higher flow rate required (assuming the same membrane area) and increased scaling tendency [58]. Typically, the tradeoff between decreasing CP via increased flow and increased recovery is one of techno-economics: higher feed flow requires more pumping capacity (increased capital costs), energy consumption (increased operating costs), and other costs associated with mitigating the increased scaling potential [59]. For a purely wave-driven system where pumping-related costs are minimal, the design optimization challenge could be more focused on other system components (e.g., pressure relief valve, accumulators, the WEC itself) to optimize crossflow velocity. Thus, future work in wave desalination directed toward system optimization should include characterization of a given system under a spectrum of conditions, akin to the “safe operating window” concept introduced in [55].

### 3.2. Ramping Experiments

After determining the transport parameters from the steady-state dataset, they are applied to a model and compared against the experimental ramping data. Calculated ramp rates (ΔP/Δt) ranged from 1.7 psi s^−1^ to 100 psi s^−1^ (0.1 bar s^−1^ to 6.9 bar s^−1^). Of the twenty unique ramp rates considered, fifteen were >10 psi s^−1^ (0.7 bar s^−1^; despite there being 28 ramping conditions, some resulted in identical ramp rates), a threshold that is commonly referenced in technical manuals as the maximum allowable ramp rate during start-up operations [27,60,61]. Because ramping in this fashion significantly deviates from steady conditions, long-term impacts to the membrane from such operations have not been considered by manufacturers. Possible consequences mentioned for exceeding 10 psi s^−1^ (0.1 bar s^−1^) during start up can include membrane telescoping, damage to the fiberglass/resin shell, failure of the glue line sealing membrane leaf edges, O-ring displacement, and generalized damage to the membrane surface resulting in a shorter lifespan [27,60,61]. While physical damage to the membrane after ramping was not explicitly evaluated here, we expect that a catastrophic failure reflective of one of these proposed mechanisms would appear in the collected data in some fashion; possibly as a sudden increase in permeate salinity and/or decrease in permeate flux. Such an observation would be a valuable experimental outcome, as it would be evidence of possible consequences of these conditions, but it was not observed. However, proper design and techno-economic analysis of full-scale wave-driven desalination systems would require an understanding of how such dynamic conditions impact membrane performance and lifetime and should be the subject of future work.

**Figure 6:**
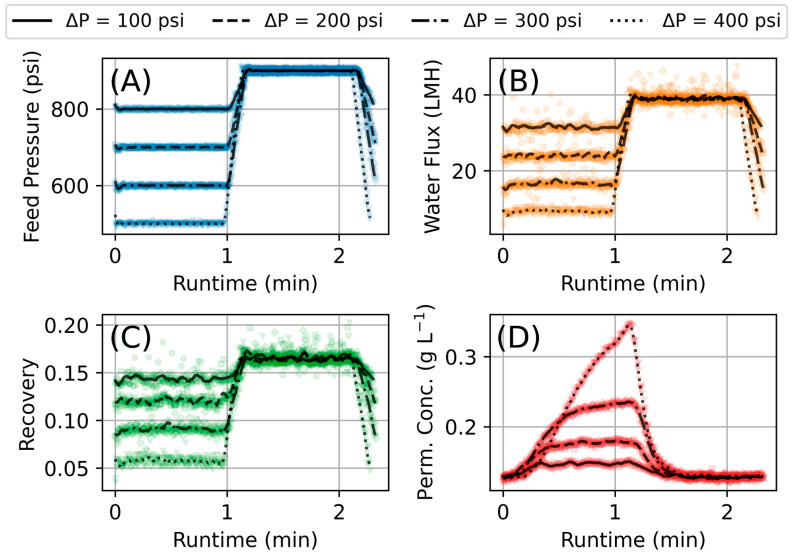
Comparison of collected (dots) to filtered (dashed lines) experimental ramping data for a single cycle with Δt = 10 s and ΔP = 100, 200, 300, 400 delineated by line style; (**A**) feed pressure, (**B**) water flux, (**C**) recovery fraction, and (**D**) permeate concentration.

As detailed in the methods section, after data collection, the data was filtered prior to making calculations necessary to obtain the transport parameters. Figure 6 shows an example of the effect of data filtering on the measured feed pressure, flux, recovery, and estimated permeate concentration. For non-conductivity measurements, the impact of the changing pressure is nearly instant; we see, e.g., the flux start increasing the moment the pressure is increased. However, conductivity measurements show very transient behavior due to the changing mass of salt in the stream having to travel to the downstream conductivity meter and the diffusive nature of dissolved salt in water.

**Figure 7:**
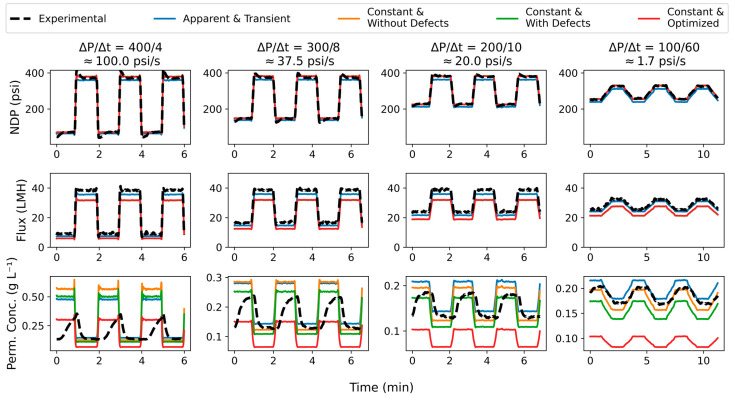
Simulation results for NDP (**top** row), $J_{w}$ (**middle** row), and $c_{p}$ (**bottom** row) for ΔP/Δt = 400/4, 300/8, 200/10, and 100/60. Experimental results are in the blacked dashed lines and results from the different parameter estimation approaches are in colored lines.

The time-series results for four select experiments are presented in Figure 7, representing a diversity of ramp rates. From left to right, the columns represent ΔP/Δt = 400/4; 300/8; 200/10; and 100/60. From top to bottom, the rows represent NDP, $J_{w}$, and $c_{p}$. The dashed black line is the experimental values for each parameter, and the colored lines represent from using the different transport parameter values determined in this study. For NDP and $J_{w}$, all of the simulations using constant parameters produced a nearly identical result, so those results appear overlaid with each other in the figure. In general, the simulation results match what might be expected under such conditions: at higher NDP, $J_{w}$ is higher and $c_{p}$ is lower. Based on visual inspection of this figure, the apparent and transient assumption produces the outcome closest to the experimental value for $J_{w}$. None of the time-series simulation results for $c_{p}$ appear to be close to the experimental result. But this is not surprising because the experimental results have an inherent time delay due to placement of the sensor. For that same reason, the $c_{p}$ simulation results are the most interesting, as we are able in a sense to get a glimpse into how the time series would appear if that delay were eliminated since the simulation is showing the behavior right at the membrane.

**Figure 8:**
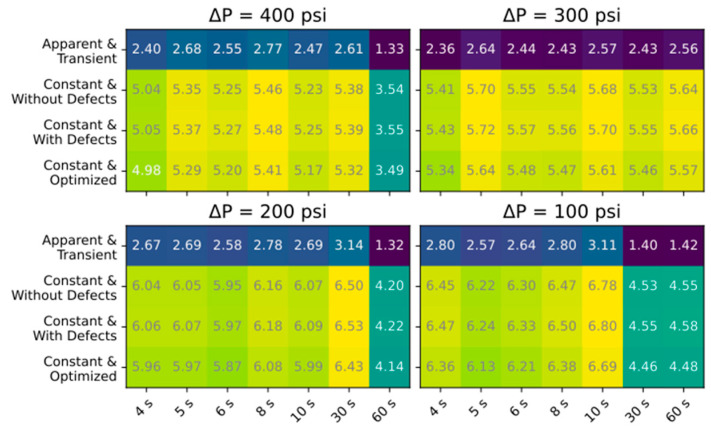
MAE of $J_{w}$ simulation results for each of the different approaches taken. Darker colors represent more accurate simulations.

**Figure 9:**
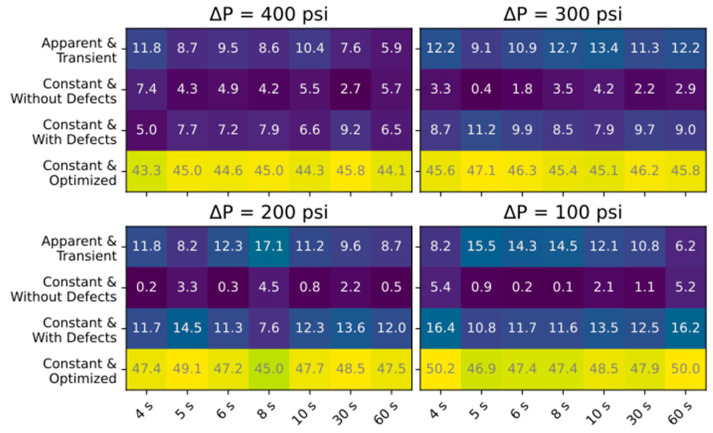
Percent difference between the simulated and experimental $c_{eff}$. Darker colors represent more accurate simulations.

Figure 8 presents the MAE of $J_{w}$ and Figure 9 presents the absolute percent error in $c_{eff}$ for the simulations of all 28 experimental conditions. The results are grouped by ΔP, with the x-axis representing Δt and the y-axis representing the different approaches used to calculate transport parameters. Feed concentration was observed to decrease ~5% between steady-state and experimental regimes due to system flushing; this would minimally impact simulation predictions but still result in valid comparisons since the effect is across all ramping experiments. For $J_{w}$, the results show that using apparent and transient transport parameters produced the most accurate simulations of the different parameter combinations, particularly for slower ramp rates. The rest of the approaches resulted in similar MAE, with slightly lower MAEs for the longest ramp rates. For the $c_{eff}$ error presented in Figure 9, assuming constant parameters without defects produced the closest results, though the estimations without defects and assuming apparent and transient are also close. A limitation in comparing simulated to experimental $c_{eff}$ is that due to the delay in conductivity measurement, the beginning of the modeled time period reflects higher pressures (and therefore lower conductivity measurements), resulting in lower estimated $c_{eff}$, and this effect is enhanced for shorter ramp times. This is less of an issue in the comparative analysis presented here (since this error would be reflected in all the comparisons), but accurately predicting instantaneous permeate concentration would require properly characterizing the residence time distribution for the permeate channel, collection tube, and process tubing between the membrane outlet and the conductivity sensor. Though this was beyond the scope of this work, simplifying assumptions can be made, as detailed in [43].

As discussed previously, discrepancies between the modeled and experimental values could be because of the known error in water and salt permeability calculations introduced by the simplifying assumptions of the solution–diffusion model commonly used (and used in this work). While typically one would not intentionally introduce error into a model, it could be a more accessible approach to modeling RO systems for wave desalination given the potentially large pressure and flow fluctuations they may experience. Interestingly, the approach employed in this work of collecting operational data over a range of pressures is the suggested experimental approach to incorporating both advection and diffusion into the model transport parameters [29] (though we did not do that). While such rigorous analysis would lead to more accurate transport parameters, creating a “calibration curve,” so to speak (as we did in essence in to test the apparent and transient assumption) for a specific system could be a more straightforward approach to obtaining a sufficiently accurate result. So, while using constant parameters may be a more conventional approach to modeling RO systems (i.e., assuming constant transport parameters across all conditions), using such an approach may not account for the inherent error in measuring water and salt permeabilities at the low operating pressures that might be experienced by wave systems. Accurately predicting water production and product salinity are critical for conducting adequate techno-economic analysis and performing resource assessments. A suggested method from [29] for determining water and salt permeability given the limitations of the solution–diffusion model is to calculate A using a high operating pressure and calculate B at low operating pressure. While such a two-point approach could produce acceptable results for a steady-state operating system, a dynamic system like wave-powered desalination could benefit from a finer relationship across all operating pressures. The utility of this approach for a wave desalination system should be examined in future research.

## 4. Conclusions

This study sought to assess if membrane transport parameters can be modeled as apparent and transient or constant under dynamic conditions like those potentially present in a wave desalination system. A primary objective was to evaluate the impact to membrane transport parameters under controlled transient conditions (ramping). The experimental results provide valuable insights into how the system responds to dynamic pressure changes. These results are relevant to not only the specific application (i.e., wave-driven desalination) but also other renewable-energy-integrated desalination systems (e.g., those with photovoltaics) that could experience similar—though likely not as drastic—pressure and flow fluctuations.

The conclusion here is not that water and salt permeability are *in fact* dynamic and transient parameters. Rather, that the value used in a solution–diffusion model can be approximated as a function of the changing inlet condition, and that this approximation can produce acceptable results in a simulation. In the context of determining values to use in a model for wave desalination, using an equation such as the form presented here could be a decent estimation. Under conventional RO operations, such an equation is meaningless since the operating conditions rarely deviate long enough to make a meaningful impact on a modeled result. Under the dynamic conditions of wave desalination, however, it is very possible that the operating window for a membrane element will span a wide range of operating pressures, including dipping below the osmotic pressure of the feed water. Being able to accurately model the RO system under these conditions could help in designing the wave-energy converters, assessing potential resources/locations for wave desalination systems, and give more accurate results regarding the product water salinity and volume produced over a given period of time.

Properly characterizing both the advective and diffusive transport coefficients requires data collection at several pressures. A key finding from the experiments is that the transient representation of apparent water and salt permeability of the RO system had as much predictive power as the constant assumption. The approach used here could be taken by others engaged in wave-powered desalination to build predictive models for their specific system.

This study, in tandem with other recently published investigations, provides more evidence that, despite not being designed for such an application, conventional and commercially available desalination membranes can provide high salt rejection and consistent water production under these variable conditions. To advance this integrated system toward commercialization, future work should consider other typical challenges faced by conventional steady-state desalination systems including proper pretreatment and brine management strategies, as well as understanding water and salt mass transfer dynamics. Finally, understanding and characterizing mechanisms of fouling and scaling under these conditions will be critical to performing a techno-economic analysis of these systems and developing mitigation strategies to improve membrane lifetime.

## Figures and Tables

**Table 1:** Assumed parameters for the membrane and feed solution.

Property	Value	Units	Reference
Membrane Active Area	7.4	m^2^	[35]
Spacer Thickness ^1^	28	mil	[35]
Spacer Porosity	0.89	-	[36]
Kinematic Viscosity ν	1.07 × 10^−6^	m^2^ s^−1^	[37,38]
$\mathrm{Diffusivity}\;D_{NaCl}$	1.32 × 10^−9^	m^2^ s^−1^	[37,38]
$\mathrm{Feed}\;\mathrm{Density}\;\rho_{f}$	1025	kg m^−3^	[37,38]
$\mathrm{Permeate}\;\mathrm{Density}\;\rho_{p}$	998	kg m^−3^	[37,38]

^1^ The spacer thickness is assumed to be the height of the channel $h_{chan}.$

**Table 2:** Summary of steady-state experiment results.

Pressure psi	Net Driving Pressure psi	Water Permeability LMH psi^−1^ (LMH bar^−1^)	Salt Permeability LMH	Feed Flow Rate L min^−1^	Flux LMH	Water Recovery %	Salt Rejection %	Effective Permeate Conc. mg L^−1^
500	39.2 ± 0.3	0.121 (1.75)	0.090	16.2 ± 0.1	4.7 ± 0.5	3.0 ± 0.4	98.1	770
600	107.3 ± 0.1	0.109 (1.58)	0.102	18.4 ± 0.1	11.7 ± 0.5	6.6 ± 0.3	99.1	375
700	182.7 ± 0.1	0.104 (1.51)	0.108	20.4 ± 0.1	18.9 ± 0.5	9.6 ± 0.2	99.4	260
800	259.3 ± 0.1	0.100 (1.45)	0.116	22.3 ± 0.1	25.9 ± 0.5	12.0 ± 0.5	99.5	210
900	332.7 ± 0.1	0.099 (1.44)	0.119	24.1 ± 0.1	32.9 ± 0.5	14.2 ± 0.3	99.6	180

**Table 3:** Coefficients for water and salt permeability fit equations.

Fit Parameter	Value
$a_{1}$	0.03528
$a_{2}$	0.01085
$a_{3}$	0.09831
$b_{1}$	0.01331
$b_{2}$	0.15060
$b_{3}$	0.06588

**Table 4:** Summary of water and salt permeability relationships developed using steady-state data.

Processing Approach	Water Permeability	Salt Permeability
Apparent and Transient	$a_{1}\exp\left(-a_{2}NDP\right)+a_{3}$	$b_{1}\ln\left(b_{2}NDP\right)+b_{3}$
Constant and Without Defects	0.083 LMH psi^−1^ (1.20 LMH bar^−1^)	0.093 LMH
Constant and With Defects	0.083 LMH psi^−1^ (1.20 LMH bar^−1^)	0.082 LMH
Constant and Optimized	0.084 LMH psi^−1^ (1.22 LMH bar^−1^)	0.049 LMH

## Data Availability

The original data used for the study are openly available on the Marine and Hydrokinetic Data Repository as Submission 649 (https://mhkdr.openei.org/submissions/649, accessed on 30 July 2025) [62].

## Declaration

The views expressed in the article do not necessarily represent the views of the DOE or the U.S. Government. The U.S. Government retains and the publisher, by accepting the article for publication, acknowledges that the U.S. Government retains a nonexclusive, paid-up, irrevocable, worldwide license to publish or reproduce the published form of this work, or allow others to do so, for U.S. Government purposes.

## Abbreviations

The following abbreviations are used in this manuscript:

CP	Concentration Polarization
CPF	Concentration Polarization Factor
LMH	Liters per meter squared per hour
MAE	Mean Absolute Error
NDP	Net Driving Pressure
RO	Reverse Osmosis
TMP	Transmembrane Pressure
WEC	Wave-Energy Converter
